# Impact of Comorbid Sleep-Disordered Breathing and Atrial Fibrillation on the Long-Term Outcome After Ischemic Stroke

**DOI:** 10.1161/STROKEAHA.123.042856

**Published:** 2024-01-26

**Authors:** Xiaoli Yang, Julian Lippert, Martijn Dekkers, Sebastien Baillieul, Simone B. Duss, Tobias Reichlin, Anne-Kathrin Brill, Corrado Bernasconi, Markus H. Schmidt, Claudio L.A. Bassetti

**Affiliations:** 1 Department of Neurology (X.Y., J.L., M.D., S.B.D., C.B., M.H.S., C.L.A.B.), Inselspital, Bern University Hospital, University of Bern, Switzerland.; 2 Interdisciplinary Sleep-Wake-Epilepsy-Center (X.Y., J.L., M.D., S.B.D., A.-K.B., M.H.S., C.L.A.B.), Inselspital, Bern University Hospital, University of Bern, Switzerland.; 3 Department of Cardiology (T.R.), Inselspital, Bern University Hospital, University of Bern, Switzerland.; 4 Department of Pulmonary Medicine and Allergology (A.-K.B.), Inselspital, Bern University Hospital, University of Bern, Switzerland.; 5 Grenoble Alpes University, HP2 Laboratory, INSERM U1300 and Grenoble Alpes University Hospital, France (S.B.).

**Keywords:** atrial fibrillation, heart failure, hypertension, ischemic stroke, stroke

## Abstract

**BACKGROUND:**

Sleep-disordered breathing (SDB) and atrial fibrillation (AF) are highly prevalent in patients with stroke and are recognized as independent risk factors for stroke. Little is known about the impact of comorbid SDB and AF on long-term outcomes after stroke.

**METHODS:**

In this prospective cohort study, 353 patients with acute ischemic stroke or transient ischemic attacks were analyzed. Patients were screened for SDB by respiratory polygraphy during acute hospitalization. Screening for AF was performed using a 7-day ECG up to 3× in the first 6 months. Follow-up visits were scheduled at 1, 3, 12, 24, and 36 months poststroke. Cox regression models adjusted for various factors (age, sex, body mass index, hypertension, diabetes, dyslipidemia, and heart failure) were used to assess the impact of comorbid SDB and AF on subsequent death or cerebro-cardiovascular events.

**RESULTS:**

Among 353 patients (299 ischemic stroke and 54 transient ischemic attacks), median age, 67 (interquartile range, 57–74) years with 63% males. Moderate-to-severe SDB (apnea-hypopnea index score, ≥15/h) was present in 118 (33.4%) patients. Among the 56 (15.9%) patients with AF, 28 had comorbid moderate-to-severe SDB and AF. Over 36 months, there were 12 deaths and 67 recurrent cerebro-cardiovascular events. Patients with comorbid moderate-to-severe SDB and AF had a higher risk of subsequent death or cerebro-cardiovascular events compared with those with only moderate-to-severe SDB without AF (hazard ratio, 2.49 [95% CI, 1.18–5.24]) and to those without moderate-to-severe SDB or AF (hazard ratio, 2.25 [95% CI, 1.12–4.50]). However, no significant difference was found between the comorbid moderate-to-severe SDB and AF group and the group with only AF without moderate-to-severe SDB (hazard ratio, 1.64 [95% CI, 0.62–4.36]).

**CONCLUSIONS:**

Comorbid moderate-to-severe SDB and AF significantly increase the risk of long-term mortality or recurrent cerebro-cardiovascular events after acute ischemic stroke. Considering both conditions as cumulative and modifiable cerebro-cardiovascular risk factors is of interest for the management of acute stroke.

**REGISTRATION:**

URL: https://www.clinicaltrials.gov; Unique identifier: NCT02559739.

Sleep-disordered breathing (SDB) is found in over 50% of patients with acute but also subacute and chronic stroke.^1,2^ Among stroke conditions, approximately one-third exhibit severe SDB with an apnea-hypopnea index (AHI) score of ≥30/h. The association of SDB and stroke is detrimental, as SDB independently increases the risk of stroke recurrence,^3^ as well as mortality and impairs functional recovery after stroke.^4^

AF is the most common sustained cardiac arrhythmia in patients with ischemic stroke, with 20% to 30% having known AF.^5^ It is associated with a 4- to 5-fold increase in incident stroke risk,^6^ and ≈15% of all strokes are caused by AF. AF is associated with longer stays in hospital, greater disability, higher rates of stroke recurrence and mortality compared with patients with stroke without AF.^7^

Early observational investigations indicated that moderate-to-severe SDB (AHI score, ≥15/h) is present in up to 21.5% of patients with AF,^8^ whereas other data suggest that as many as 74% of patients with AF may have SDB (AHI score, >10/h).^9^ In comparison to those without SDB (AHI score, <5/h), individuals with moderate-to-severe SDB were reported to be 2 to 4× more likely to develop AF and the risk of AF is strongly associated with SDB severity.^10^ Furthermore, the relationship between these 2 conditions remains even after controlling for confounding conditions such as systemic hypertension, obesity, and heart failure.^11^

Long-term exposure to apneic events was shown to be linked to structural remodeling processes which increase AF-inducibility.^12,13^ Interestingly, growing evidence suggests that nocturnal AF paroxysms are not solely explained by structural changes in the atrial myocardium but acute transient arrhythmogenic changes during apneas may further contribute to AF development.^14,15^

The association between AF and SDB appears to be complex and bidirectional. These findings lend support to the hypothesis that the cause of AF may involve a distinctive interplay between the underlying pathophysiological mechanisms of SDB and AF.

Despite the evidence of epidemiological and pathological mechanisms linking SDB, AF, and cerebro-cardiovascular events (CCVE), there is, to our knowledge, no study investigating the poststroke association of SDB with AF and the impact of this combination on the long-term outcome. To gain insights into this topic, the present study was designed to investigate the impact of comorbid SDB and AF on the risk of recurrent CCVE including all-cause mortality in patients with ischemic stroke/TIA. We hypothesized that comorbid SDB and AF should be associated with an increased risk of recurrent CCVE including all-cause mortality in patients with ischemic stroke/TIA.

## METHODS

### Data Availability

The data that support the findings of this study are available from the corresponding author upon reasonable request.

### Study Design and Participants

This study is based on the Sleep Deficiency and Stroke Outcome Study (https://www.clinicaltrials.gov; NCT02559739), a prospective observational cohort study that was designed to investigate the impact of sleep deficiency and sleep fragmentation on the short- and long-term outcome of ischemic stroke and TIA. The sleep and stroke teams at the Bern University Hospital (recruitment period, July, 2015 to January, 2018) and the Neurocenter of Southern Switzerland of the Ospedale Regionale di Lugano (recruitment period, November, 2015 to July 2016) recruited patients with acute ischemic stroke or TIA consecutively admitted to their stroke centers. Follow-up visits were conducted at 1, 3, 12, 24, and 36 months after stroke onset. For this analysis, only patients admitted to the Bern University Hospital were included, as they had consistently available long-term ECG data and were followed up over a total time period of 36 months. The study was approved by the local ethics committees of Bern and Lugano, Switzerland. Eligible patients were 18 to 85 years of age and able to provide written informed consent.

Exclusion criteria were primary hemorrhagic stroke, clinically unstable or life-threatening conditions (eg, coma/stupor, severe heart failure, and persisting oxygen dependency), pregnancy, drug or alcohol abuse, and incapability to follow the study procedures.

### Polygraphic, Medical Recordings, and 7-Day ECG

Within 3 days after admission (at the latest 7 days after an acute stroke/TIA), 190 (54%) patients were screened for SDB by using a respiratory polygraphy (Nox Medical, Inc, Reykjavik, Iceland) and 163 (46%) patients with an ApneaLink (ResMed, Bella Vista, Australia).^16^

Apnea was defined as a ≥90% reduction in nasal pressure signal, while hypopnea was defined as a ≥30% reduction in airflow combined with ≥3% arterial oxygen desaturation, both lasting at least 10 s. Results were assessed by an experienced scorer following American Academy of Sleep Medicine guidelines.^17^ SDB presence was defined as AHI score of ≥5/h and categorized as mild (AHI score, 5–<15/h), moderate (AHI score, 15–<30/h), or severe (AHI score, ≥30/h).^17^

Screening for AF during the acute phase was performed with continuous ECG monitoring. In case of negative results during the ECG monitoring, ambulatory 7-day ECG was repeated up to 3× during the first 6 months after the stroke. According to the current consensus definition of paroxysmal AF, an arbitrary cut-off of ≥30 s was applied.^18^ For analysis, both patients with baseline AF and AF detected during follow-up were considered.

The following additional information was collected: demographics, medical history, stroke/TIA characteristics and severity at both hospital admission and discharge using the National Institutes of Health Stroke Scale,^19^ and stroke etiology based on the TOAST study (Trial of the ORG 10172 in Acute Stroke Treatment).^20^

### Long-Term Outcome: All-Cause Mortality and Incident CCVE After 36 Months

The primary end point was a composite of death from any cause, stroke (ischemic and hemorrhagic), TIA, nonfatal myocardial infarction, unplanned hospitalization (or unplanned prolongation of a planned hospitalization) for heart failure, urgent revascularization, or other vascular events within the first 36 months after the acute event.

Events were assessed through structured telephone interviews conducted by research fellows or study nurses. Patients’ general practitioners/attending physicians were contacted to collect the primary end point if neither patients nor surrogates were unable to provide appropriate information. Survival status was also obtained by querying the Central Compensation Office, which registers all deaths of Swiss citizens or individuals residing in Switzerland, through the hospital’s patient management system. In case of missing information regarding the outcome of interest, we assumed that no event occurred. Subsequent recurrent events in individual patients were not considered.

### Statistics

Descriptive statistics summarized demographics and baseline data, including counts, percentages, median, and interquartile range. Continuous and categorical variables were compared using appropriate tests: Mann-Whitney *U* test, Pearson χ² test, or Fisher exact test.

Univariate Cox regression assessed each baseline variable’s impact on all-cause mortality and recurrent CCVE using log-transformed AHI and dichotomous AHI (cutoffs, 5, 15, and 30/h). Subsequently, partly adjusted (each SDB metric+AF independently) and fully adjusted Cox regression analyzed comorbid moderate-to-severe SDB and AF, along with the differential impact of SDB and AF on the long-term outcome, adjusting for age, sex, body mass index (BMI), hypertension, diabetes, dyslipidemia, and heart failure. Multivariate logistic regression tested the association between moderate-to-severe SDB and AF occurrence, adjusting for the aforementioned covariates.

To test the association between stroke subtypes and the coexistent moderate-to-severe SDB and AF, multivariate logistic regression was used with adjustment for age, sex, and hypertension. Kruskal-Wallis test was performed to compare the initial stroke severity in patients with comorbid moderate-to-severe SDB and AF (SDB^+^/AF^+^), only moderate-to-severe SDB without AF (SDB^+^/AF^−^), only AF without moderate-to-severe SDB (SDB^−^/AF^+^) and neither moderate-to-severe SDB nor AF (SDB^−^/AF^−^) (AHI cutoff score: 15/h).

Supportive analyses explored changing explanatory variables in Cox regression models. Additional analyses examined the interaction effect of SDB and AF on long-term outcomes. Multicollinearity was checked using variance inflation factors without imputation for missing values.

The planned sample size for this study was ≈520 patients, a number justified by the need to assess the prognostic value of individual sleep disorders in relation to CCVE. During the predetermined recruitment period, a total of 395 patients were evaluable for this analysis (with 353 contributing to the key regression analysis), which is still considered sufficient, especially on the basis of the number of primary events.

Statistical significance was defined as *P*<0.05. All analyses were conducted in R version 4.0.2 (R Foundation for Statistical Computing; www.r-project.org). This study adheres to the Strengthening the Reporting of Observational Studies in Epidemiology guideline.^21^

## RESULTS

Among the 395 patients included in the study with acute ischemic stroke or TIA, 42 patients were excluded because of either missing sleep test (n=41) or 7-day ECG monitoring (n=1; Figure S1). A total of 353 patients were finally analyzed. Excluded patients did not differ from the analyzed population regarding age, sex, BMI, and initial stroke severity (Table S1). For follow-up visits, 326 (83%) patients were contacted after 1 month, 332 (84%) after 3 months, 318 (81%) after 12 months, 310 (78%) after 24 months, and 287 (73%) after 36 months.

Baseline study population consisted of 353 patients with 299 (85%) having an ischemic stroke and 54 (15%) a TIA with a median National Institutes of Health Stroke Scale score of 2.0 (interquartile range, 1–4). The median AHI was 7.8/h (interquartile range, 3.5–20.0/h); 118 (33%) patients were diagnosed with a moderate-to-severe SDB (AHI score, ≥15/h) and 50 (14%) patients had severe SDB (AHI score, ≥30/h). Thirty-two (9%) patients had preknown SDB. Thirty-five (10%) patients had a history of AF, while in 21 (6%) patients AF was detected after stroke.

Forty-five (80%) of these 56 patients with AF (including baseline and follow-up) received oral anticoagulation, 5 patients received antiplatelet therapy, and data were not available in 6 patients. During the follow-up visits, continuous positive airway pressure (CPAP) therapy was received by only 18 (15%) patients of 118 moderate-to-severe patients with SDB. Among them, 6 patients had initiated CPAP before the occurrence of stroke/TIA, while 12 patients initiated the treatment during the follow-up period. Only 8 (44%) of 18 patients continued treatment 12 months after stroke/TIA onset.

Patients with stroke with AF were relatively older, more often male, had a higher AHI, higher frequency of prestroke hypertension, and a higher occurrence of cardioembolic stroke compared with those without AF (Table 1). Of all 56 patients diagnosed with AF, 28 patients (50%) had comorbid moderate-to-severe SDB and AF (Table 2).

**Table 1.**
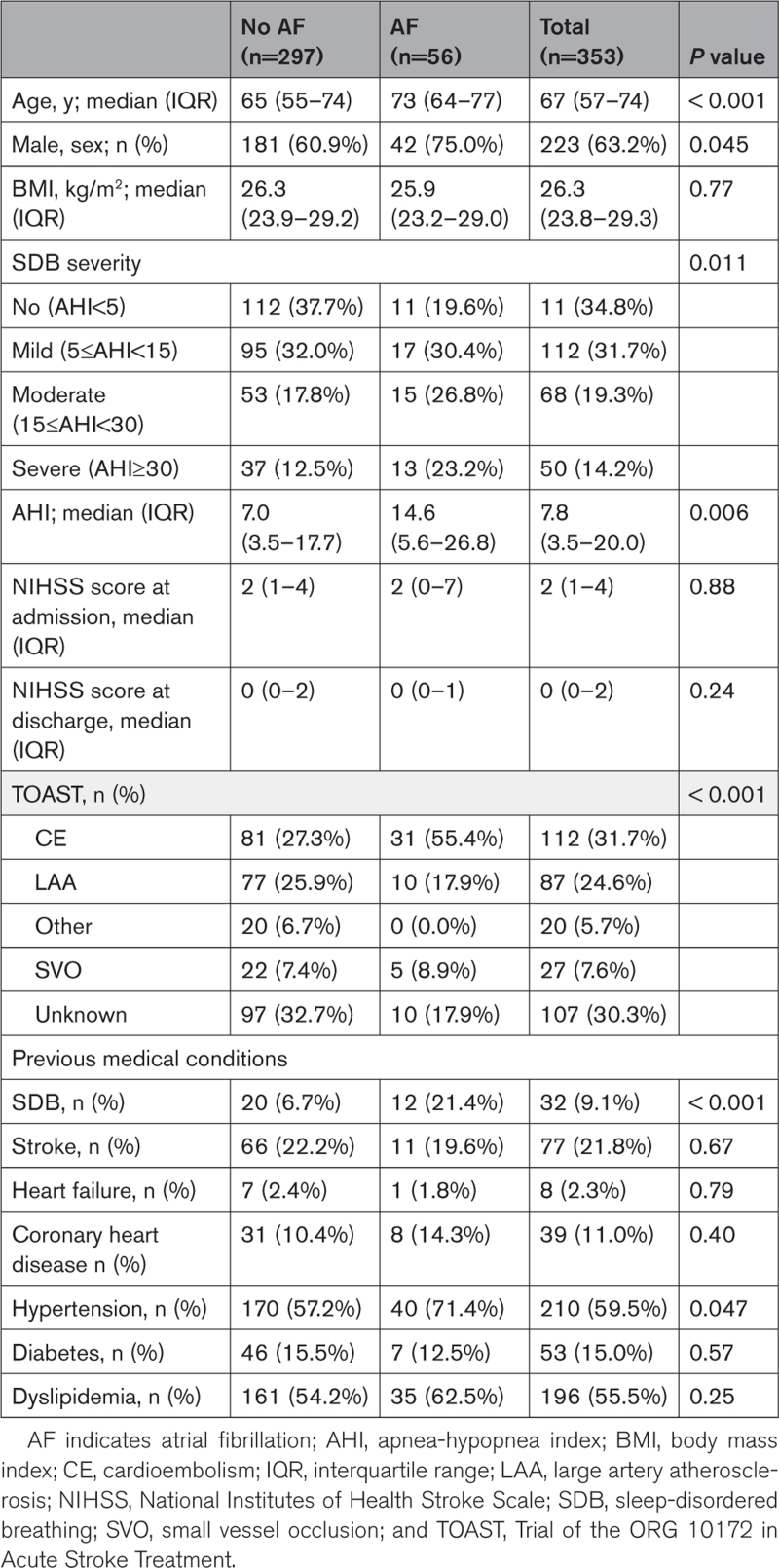
Patient Characteristics at Baseline Stratified by AF

**Table 2.**
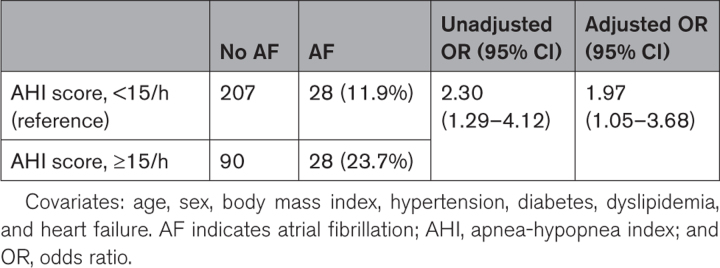
Frequency of AF in AHI Score, ≥15 Versus <15/h

**Figure 1.**
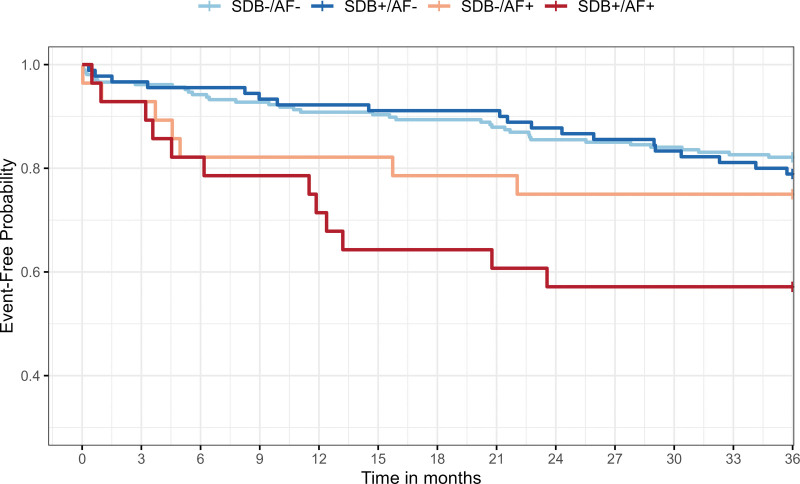
**Event-free Kaplan-Meier curves for the risk of death or recurrent cerebro-cardiovascular events in SDB^−^/AF^−^, SDB^+^/AF^−^, SDB^−^/AF^+^, and SDB^+^/AF^+^ groups during the 36-mo follow-up.** SDB^−^/AF^−^: Apnea-Hypopnea Index (AHI)<15/h without AF (n=207); SDB^+^/AF^−^: AHI≥15/h without AF (n=90); SDB^−^/AF^+^: AHI<15/h with AF (n=28); SDB^+^/AF^+^: AHI≥15/h with AF (n=28). Covariates: age, sex, body mass index, hypertension, diabetes, dyslipidemia, and heart failure. AF indicates atrial fibrillation; and SDB, sleep-disordered breathing.

During the 36 months’ follow-up, 67 (19%) recurrent patients with CCVE were reported and 12 (3%) patients were died during the observational period. The most frequently observed CCVE was recurrent stroke, 25 (7%) and TIA, 17 (5%). In addition, 8 subsequent heart failure leading to hospitalization, 3 myocardial infarction, 2 events of unstable angina requiring urgent revascularization, and 12 other vascular events were recorded. Multivariate Cox regression showed that patients with stroke/TIA with SDB^+^/AF^+^ had a higher risk of death or recurrent CCVE than patients with SDB^+^/AF^−^ (hazard ratio, 2.49 [95% CI, 1.18–5.24]) or SDB^−^/AF^−^ (hazard ratio, 2.25 [95% CI, 1.12–4.50]) after accounting for age, sex, BMI, hypertension, diabetes, dyslipidemia, and heart failure. In contrast, no significant association was observed between SDB^+^/AF^+^ and SDB^−^/AF^+^ (AHI score, <15/h; hazard ratio, 1.64 [95% CI, 0.62–4.36]; Figure 1).

**Figure 2.**
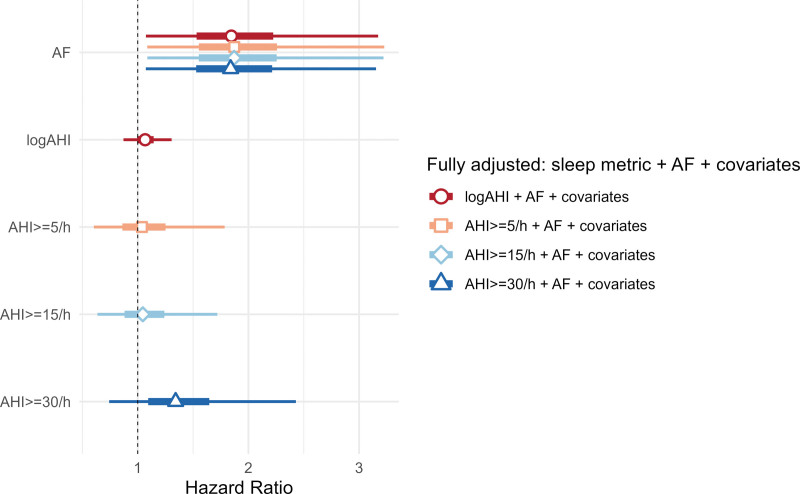
**Hazard ratio of incident death or cerebro-cardiovascular events after 36 mo visits.** AF indicates atrial fibrillation; and AHI, Apnea-Hypopnea Index.

To further characterize the differential impact of SDB and AF on the long-term outcome of stroke and TIA, univariate Cox regression was first performed in log-transformed AHI, dichotomized AHI with cutoff at 5, 15, and 30/h, and AF. Significant HRs for AF, log-AHI, and AHI score of ≥30/h were observed. Then, partly and fully adjusted Cox regression models were used. Although the severe SDB group approached significance in model 2d implicating SDB as an independent risk for CCVE, the associations between SDB and long-term outcome in both partly adjusted models (AF+sleep metrics) and fully adjusted models became nonsignificant. However, the impact of AF persisted and remained statistically significant after adjusting for all covariates (Figure 2; Table S2). No significant interaction was found between SDB and AF, regardless of whether SDB was considered as a categorical variable (AHI score, ≥15/h; *P*=0.23) or when examining log-transformed AHI (*P*=0.89).

In addition, we tested the association between AF and SDB (AHI score, ≥15/h). Multivariate logistic regression shows that AF was significantly more frequent in SDB after adjusting for age, sex, BMI, hypertension, diabetes, dyslipidemia, and heart failure (AHI score, ≥15/h versus AHI score, <15/h; odds ratio, 1.97 [95% CI, 1.05–3.68]; Table 2).

**Figure 3.**
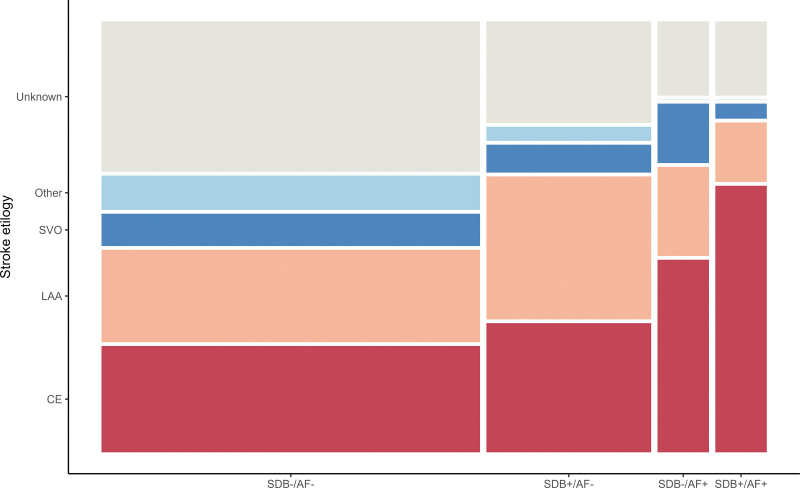
**The mosaic plot of the distribution of stroke etiology in SDB^−^/AF^−^, SDB+/AF^−^, SDB^−^/AF+, and SDB+/AF+ groups.** Caption: 231, 66, 33, and 23 patients are in SDB^−^/AF^−^, SDB+/AF^−^, SDB^−^/AF+, and SDB+/AF+ groups, separately. The frequencies of cardioembolism (CE) are 61, 20, 16, and 15, separately. The frequencies of large artery atherosclerosis (LAA) are 50, 27, 8, and 2, respectively. AF indicates atrial fibrillation; SDB, sleep-disordered breathing; and SVO, small vessel occlusion.

Next, we studied the association between SDB, AF, and stroke subtypes. Using the TOAST classification, the highest proportion of cardioembolic stroke was observed in the SDB^+^/AF^+^ group, whereas a relatively lower proportion was observed in the SDB^−^/AF^+^ group (65% versus 48%; Figure 3). The highest risk of cardioembolic stroke was observed in the SDB^+^/AF^+^ group compared with the SDB^−^/AF^+^ group and compared with the SDB^−^/AF^−^ group (SDB^+^/AF^+^ versus SDB^−^/AF^−^: odds ratio, 6.20 [95% CI, 2.64–15.35]; SDB^−^/AF^+^ versus SDB^−^/AF^−^: odds ratio, 2.68 [95% CI, 1.17–6.13]). Moreover, the SDB^+^/AF^−^ group had a significantly higher frequency of large artery atherosclerosis stroke than the SDB^−^/AF^−^ group (odds ratio, 1.96 [95% CI, 1.06–3.59]) after accounting for age, sex, and hypertension.

Finally, we investigated the associations between SDB, AF, and stroke severity at admission. No significant difference in initial National Institutes of Health Stroke Scale scores was observed between these 4 groups (*P*=0.10).

## DISCUSSION

This study assessed for the first time the impact of comorbid SDB and AF on the long-term outcome in patients with ischemic stroke and TIA and offered 3 main results. First, patients with stroke and TIA with comorbid moderate-to-severe SDB and AF have worse long-term cerebro-cardiovascular outcomes and higher mortality compared with patients with only moderate-to-severe SDB or with neither moderate-to-severe SDB nor AF, independent of age, sex, BMI, hypertension, diabetes, dyslipidemia, and heart failure. Second, AF is the dominant risk factor for recurrent CCVE or mortality, as moderate-to-severe SDB alone did not significantly increase CCVE. However, it is important to note that moderate-to-severe SDB is known to significantly increase the occurrence of AF, consistent with our results showing that patients with moderate-to-severe SDB had a significantly increased prevalence of AF. Third, comorbid moderate-to-severe SDB and AF are associated with a higher cardioembolic stroke mechanism.

The study highlights the role of AF as a potential major stroke mechanism in patients with SDB. These findings are in line with a prospective study of 134 patients showing both a high prevalence of SDB in stroke patients and identifying AF as a dominant predictor of stroke in patients with SDB.^22^

Both SDB and AF share common risk factors and several pathophysiological mechanisms link SDB to AF such as significant fluctuations in autonomic tone during obstructive apneic events,^23^ increased systemic inflammation, atrial fibrosis, as well as atrial enlargement and remodeling.^24,25^

To date, only a few studies have investigated the impact of SDB on cerebral and cardiovascular mortality and morbidity with adjusting for AF.^26–28^ Ponsiang et al found that a high AHI score (>24/h) no longer affected survival in patients with stroke when adjusted for the effects of age, disability, and AF, whereas there was a trend toward 3.63× higher mortality risk with AF,^26^ which is in line with our results showing that patients with AF doubled the mortality risk compared with patients without AF. Besides, our study indicates a higher prevalence of AF in moderate-to-severe SDB. These findings are in accordance with results from a previous large population-based study of 6841 patients showing that SDB severity is independently associated with incident AF.^10^

Results from previous studies investigating the direct impact of SDB on long-term outcomes are heterogeneous. Most of these studies focus almost exclusively on AHI to quantify the severity of SDB. A meta-analysis of prospective clinical and population-based studies suggests SDB as an independent stroke predictor.^29^ Further studies describe an independent impact of SDB on mortality^30,31^ and recurrent CCVE.^32^ Moreover, in a long-term follow-up study with 842 patients followed for 591 days, Brown et al^3^ showed that SDB is associated with recurrent ischemic stroke independent of demographics, BMI, cardiovascular risk factors, and initial stroke severity. Contrary, a large decade-long cohort study reports that not AHI alone but SDB-related factors such as sleep time spent with oxygen saturation <90%, number of awakenings, mean heart rate, or excessive daytime sleepiness are significantly independently associated with an increased risk of CCVE.^33^

After adjusting for potential confounders and risk factors including AF, we also did not find SDB based on the AHI as an independent risk factor for CCVE and mortality.

Concerning stroke subtypes, inconsistent findings have been published on the frequency of cardioembolic stroke in patients with SDB. One retrospective study found a significant association between cardioembolic strokes in patients with moderate-to-severe SDB,^34^ whereas Brown et al^35^ did not find an association between SDB and stroke subtypes. Results from our study indicate a significant association between moderate-to-severe SDB and large artery origin of stroke after adjustment for age, sex, and hypertension, whereas AF is significantly associated with cardioembolism strokes as previously reported in numerous studies.^33,36^

Importantly, our study shows that the highest proportion of cardioembolism strokes was observed in the group of patients with stroke with coexistent moderate-to-severe SDB and AF. Prior research described a pathophysiological link between SDB, AF, and left atrial enlargement,^37^ as well as the association of left atrial volume with cardioembolism and AF.^38^ Although previous attempts to identify an interaction between these 2 factors yielded unfavorable results, it is essential to acknowledge that the analysis might not be conclusive due to the limitations in sample size. Future investigations are warranted to delve into the effects of SDB, not only as a direct risk factor for AF, but also as a crucial intermediary risk for cardiostructural and metabolic comorbidities.

The most important strengths of our study are the regular long-term clinical follow-up visits of up to 36 months and the prolonged sequential 7-day ECG monitoring applied up to 3 times following stroke with a high probability to detect paroxysmal AF.

Our study has many limitations. First, SDB was assessed only once in the acute phase of stroke. Some studies reported a night-to-night variability in SDB severity.^39^ After a stroke, most studies suggested that SDB following stroke/TIA remains frequent even at 3 months or later.^2,40^ Second, the assessment of SDB was performed with portable devices, which generally underestimates the frequency and severity of SDB in comparison to polysomnography.^41^ Third, cumulative evidence suggests that AHI, the generally recommended measurement for SDB, poorly predicts the adverse outcomes of sleep apnea, potentially because AHI does not adequately capture disease burden with respect to the depth and duration of ventilator disturbances and the concomitant oxygen desaturation. Rather than AHI, the desaturation and obstruction severity or so-called hypoxic burden defined as the event-associated area under the oxygen desaturation curve seems to improve the prediction of cerebro-cardiovascular outcome.^42,43^ Fourth, it should be noted that the study population primarily consists of patients with stroke with mild-to-moderate impairment, which was necessary to facilitate neurocognitive testing and questionnaires administration as part of our study design. However, this approach may have resulted in the exclusion of patients with aphasia and more severe impairments, potentially introducing a selection bias. Consequently, the generalizability of our study findings may be impacted. It is worth highlighting that the significance of SDB in influencing outcomes is underscored in this specific patient cohort, which predominantly consists of mildly to moderately affected individuals. Additionally, being a single-site study imposes limitations on the generalizability of the findings.

Our study identifies additional limitations regarding the treatment status of SDB and AF at baseline and during the follow-up period. In relation to SDB treatment, particularly the use of CPAP, there is a lack of comprehensive data on therapy compliance and adherence, which would be essential for evaluating the sustained effects over a prolonged duration. Previous trials investigating the functional outcomes of CPAP treatment in patients with stroke have yielded inconsistent results.^1^ A meta-analysis that encompassed 10 randomized controlled trials and involved 564 patients with stroke showed an overall improvement in neurofunctional outcomes with CPAP treatment.^44^ However, it is important to note that our study was not explicitly designed to thoroughly investigate this specific effect.

In the context of AF, 80% of the patients received anticoagulant therapy in adherence to the prevailing guidelines. This proactive approach effectively mitigates the potential confounding effect of inadequate treatment, consequently safeguarding the long-term outcomes.

## Conclusions

Patients with stroke with comorbid moderate-to-severe SDB and AF have a higher risk of long-term cerebro-cardiovascular morbidity and mortality compared with patients with only moderate-to-severe SDB and patients with neither moderate-to-severe SDB nor AF. AF appears to be the dominant independent risk factor. Thus, it is important to consider both conditions as cumulative and potential modifiable cerebro-cardiovascular risk factors, because of the availability of specific treatment options. Further studies are needed to confirm our observations and to assess the utility of other measures of SDB severity (eg, nocturnal hypoxemic burden and night-to-night variability) to better understand the interrelationship between SDB, AF and cerebro-cardiovascular diseases.

## ARTICLE INFORMATION

### Acknowledgments

The authors extend their gratitude to all those who contributed to the Sleep Deficiency and Stroke Outcome Study, with a special thanks to dedicated study nurses, including Nadja Steiner, Nicole Duttweiler, Tanja Gerber, Saskia Salzmann, and Jane Frangi. Their invaluable assistance in study preparation, execution, and conducting additional assessments is highly appreciated.

### Sources of Funding

This study was supported by the Swiss National Science Foundation (SNF-320030_149752), the Swiss Heart Foundation, and a PhD fellowship from the China Scholarship Council (to Xiaoli Yang)

### Disclosures

Dr Reichlin has received grants from Abbott Laboratories, Biosense Webster, Inc, Biotronik, Boston Scientific Corporation, and Medtronic, and severs as a consultant. Dr Baillieul has received a grant from the European Respiratory Society. The other authors report no conflicts.

### Supplemental Material

STROBE Checklist

Figure S1

Tables S1–S2

## Supplementary Material
