# Supplementary material for: Impact of Comorbid Sleep-Disordered Breathing and Atrial Fibrillation on the Long-Term Outcome After Ischemic Stroke
Source: Stroke. 2024 Jan 26;55(3):586–94. doi: 10.1161/STROKEAHA.123.042856 (PMC10896199; doi:10.1161/STROKEAHA.123.042856)
Supplement: Supplementary file 1 [file str-55-586-s001.pdf]

SUPPLEMENTAL MATERIAL

Impact of comorbid sleep-disordered breathing and atrial fibrillation on the long-term outcome after ischemic stroke

Authors: Xiaoli Yang[1,2 *] M.D, Julian Lippert[1,2 *] M.D, Martijn Dekkers[1,2] M.D, Ph.D, Sebastien Baillieul[3] M.D, Ph.D, Simone B. Duss[1,2] Ph.D, Tobias Reichlin[4] M.D, Anne-Kathrin Brill[2,5] M.D, Corrado Bernasconi[1] M.D, Ph.D, Markus H. Schmidt[1,2] M.D, Ph.D, Claudio L. A. Bassetti[1,2] M.D

1. Department of Neurology, Inselspital, Bern University Hospital, University of Bern, Switzerland
2. Interdisciplinary Sleep-Wake-Epilepsy-Center, Inselspital, Bern University Hospital, University of Bern, Switzerland
3. Grenoble Alpes University, HP2 Laboratory, INSERM U1300 and Grenoble Alpes University Hospital, Grenoble, France
4. Department of Cardiology, Inselspital, Bern University Hospital, University of Bern, Switzerland
5. Department of Pulmonary Medicine and Allergology, Inselspital, Bern University Hospital, University of Bern, Switzerland

Figures

Figure S1. Flow chart of patient's selection

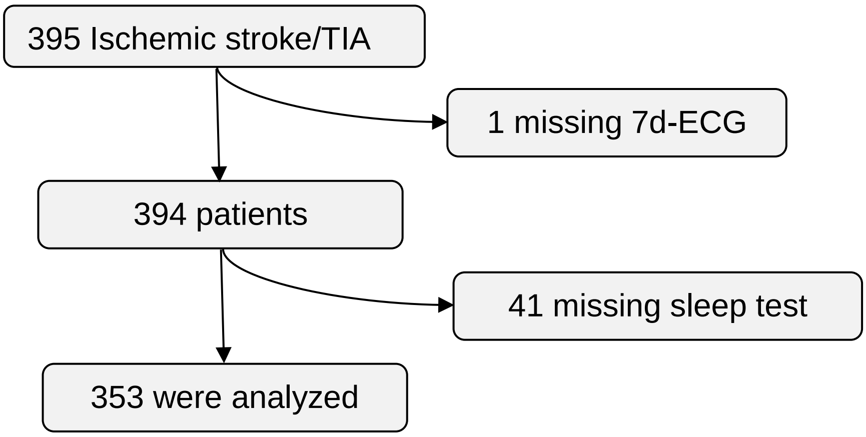

Tables

Table S1. Baseline characteristics between included and excluded patients.

	Included (n=353)	Excluded (n=42)	Total (n=395)	P value
**Age**, y; median (IQR)	67 (57-74)	68 (59-74)	70 (57-74)	0.789
**Male**, sex; n (%)	223 (63.2%)	31 (73.8%)	254 (64.3%)	0.174
**BMI**; median (IQR)	26.3 (23.8-29.3)	25.6 (24.2-30.6)	26.2 (23.9-29.4)	0.786
**NIHSS at admission** median (IQR)	2 (1-4)	2 (1-5)	2 (1-4)	0.935
**NIHSS at discharge** median (IQR)	1 (0-3)	0 (0-2)	1 (0-2)	0.034
**TOAST**; n (%)				0.405
CE	112 (31.7%)	18 (42.9%)	130 (32.9%)	
LAA	87 (24.6%)	6 (14.3%)	93 (23.5%)	
Other	20 (5.7%)	1 (2.4%)	21 (5.3%)	
SVO	27 (7.6%)	3 (7.1%)	30 (7.6%)	
Unknown	107 (30.3%)	14 (33.3%)	121 (30.6%)	
**Stroke**; n (%)	77 (21.8%)	11 (26.2%)	88 (22.3%)	0.519
**Heart failure**; n (%)	8 (2.3%)	3 (7.1%)	11 (2.8%)	0.07
**Hypertension**; n (%)	210 (59.5%)	30 (71.4%)	240 (60.8%)	0.134
**Diabetes mellitus**; n (%)	53 (15.0%)	8 (19.0%)	61 (15.4%)	0.494
**Dyslipidemia**; n (%)	196 (55.5%)	24 (57.1%)	220 (55.7%)	0.84

*Abbreviations: AHI, Apnea-Hypopnea Index; CE, Cardioembolism; LAA, Large Artery Atherosclerosis; NIHSS, National Institutes of Health Stroke Scale; SVO, Small Vessel Occlusion

Table S2. Univariate, partly adjusted, and fully adjusted Hazard Ratios for the long-term outcome using Cox proportional hazard regression.

	Univariate		Partly adjusted			Fully adjusted		
**Model 1**	**HR** (95%CI)	**P**	**Model 2**	**HR** (95%CI)	**P**	**Model 3**	**HR** (95%CI)	**P**
**AF**	1.93 (1.14-3.25)	**0.01**	Model 2a	1.81 (1.07-3.06)	**0.03**	Model 3a	1.85 (1.07-3.17)	**0.03**
			Model 2b	1.85 (1.09-3.13)	**0.02**	Model 3b	1.87 (1.09-3.23)	**0.02**
			Model 2c	1.84 (1.08-3.12)	**0.02**	Model 3c	1.87 (1.09-3.22)	**0.02**
			Model 2d	1.85 (1.09-3.13)	**0.02**	Model 3d	1.84 (1.07-3.15)	**0.03**
**LogAHI**	1.21 (1.01-1.46)	**0.04**	Model 2a	1.19 (0.99-1.42)	0.06	Model 3a	1.07 (0.87-1.31)	0.53
**AHI≥5/h**	1.42 (0.86-2.35)	0.17	Model 2b	1.32 (0.80-2.20)	0.28	Model 3b	1.04 (0.60-1.79)	0.89
**AHI≥15/h**	1.44 (0.91-2.29)	0.12	Model 2c	1.35 (0.85-2.15)	0.21	Model 3c	1.05 (0.64-1.72)	0.86
**AHI≥30/h**	1.77 (1.02-3.07)	**0.04**	Model 2d	1.67 (0.96-2.91)	0.07	Model 3d	1.34 (0.74-2.43)	0.33

***Model 2: AF + sleep metrics;**

Model 2a: AF + logAHI; Model 2b: AF + AHI (cutoff:5/h);

Model 2c: AF + AHI (cutoff:15/h); Model 2d: AF + AHI (cutoff:30/h);

†**Model 3: Model 2 + Covariates;**

Model 3a: Model 2a + Covariates; Model 3b: Model 2b + Covariates;

Model 3c: Model 2c + Covariates; Model 3d: Model 2d + Covariates;

‡**Covariates:** age, sex, BMI, hypertension, diabetes mellitus, dyslipidemia, and heart failure.
